# Neural Mechanisms Underlying the Rewarding and Therapeutic Effects of Ketamine as a Treatment for Alcohol Use Disorder

**DOI:** 10.3389/fnbeh.2020.593860

**Published:** 2020-12-10

**Authors:** Caroline E. Strong, Mohamed Kabbaj

**Affiliations:** Program in Neuroscience, Department of Biomedical Sciences, Florida State University, Tallahassee, FL, United States

**Keywords:** ketamine, alcohol use disorder (AUD), depression, addiction, mechanisms

## Abstract

Alcohol use disorder (AUD) is the most prevalent substance use disorder and causes a significant global burden. Relapse rates remain incredibly high after decades of attempting to develop novel treatment options that have failed to produce increased rates of sobriety. Ketamine has emerged as a potential treatment for AUD following its success as a therapeutic agent for depression, demonstrated by several preclinical studies showing that acute administration reduced alcohol intake in rodents. As such, ketamine’s therapeutic effects for AUD are now being investigated in clinical trials with the hope of it being efficacious in prolonging sobriety from alcohol in humans (ClinicalTrials.gov, Identifier: NCT01558063). Importantly, ketamine’s antidepressant effects only last for about 1-week and because AUD is a lifelong disorder, repeated treatment regimens would be necessary to maintain sobriety. This raises questions regarding its safety for AUD treatment since ketamine itself has the potential for addiction. Therefore, this review aims to summarize the neuroadaptations related to alcohol’s addictive properties as well as ketamine’s therapeutic and addictive properties. To do this, the focus will be on reward-related brain regions such as the nucleus accumbens (NAc), dorsal striatum, prefrontal cortex (PFC), hippocampus, and ventral tegmental area (VTA) to understand how acute vs. chronic exposure will alter reward signaling over time. Additionally, evidence from these studies will be summarized in both male and female subjects. Accordingly, this review aims to address the safety of repeated ketamine infusions for the treatment of AUD. Although more work about the safety of ketamine to treat AUD is warranted, we hope this review sheds light on some answers about the safety of repeated ketamine infusions.

## Introduction

Alcohol use disorder (AUD) is defined as a chronic relapsing brain disease arising from repeated cycles of compulsive alcohol use, withdrawal, and relapse (American Psychiatric Association, 2013; National Institute on Alcohol Abuse and Alcoholism, 2018). Even though a majority of the world’s population has consumed alcohol at one point during their lifetime, only around 15% of people develop pathological drinking patterns symptomatic of AUD, suggesting individual differences in susceptibility to AUD [ Substance Abuse and Mental Health Services Administration (SAMHSA), (2015)]. Within this subset of individuals, relapse rates increase after long periods of alcohol abstinence since 60–75% of individuals relapse after 1-year, up to 80% after 3-years, and 90% after 4-years of attempted sobriety (Polich et al., 1981; Miller et al., 2001; Moos and Moos, 2006; Evren et al., 2010). These high relapse rates result from both low treatment-seeking, given that only around 8% of the 15 million adults suffering from AUD in the United States received any form of treatment in 2018, and the fact that currently available pharmacological treatment options are ineffective in the maintenance of long term sobriety (National Institute on Alcohol Abuse and Alcoholism, 2018; Carvalho et al., 2019).

Along with many neuropsychiatric disorders, AUD treatment yields the best results when specific psychosocial and pharmacological interventions are combined (Kranzler and Sakoya, 2018; Carvalho et al., 2019). Psychosocial interventions are more effective than pharmacological interventions given that only two of the top 10 effective treatment options were pharmacological including acamprosate, a GABA_A_ receptor agonist, and naltrexone, a μ-opioid receptor antagonist (Miller and Wilbourne, 2002). Even within these effective pharmacological treatment options, efficacy is modest. A meta-analysis examining seven studies found no treatment effect when investigating the effect of acamprosate on heavy alcohol drinking (Jonas et al., 2014). The same meta-analysis only showed naltrexone to effectively reduce heavy drinking when orally administered at 50 mg but not when given orally at 100 mg or injected (Jonas et al., 2014). Even within studies testing 50 mg oral naltrexone, results were highly variable since around half of the studies analyzed showed no significant effect of treatment (Jonas et al., 2014). Finding effective treatments for AUD is compounded by methodological challenges that can significantly alter the assessment of treatment efficacy (Klemperer et al., 2018). For example, while the meta-analysis conducted in Jonas et al. (2014) showed only a modest treatment effect in one naltrexone-administered group across randomized clinical trials, another meta-analysis examining naltrexone’s treatment efficacy within human laboratory studies showed a more significant treatment outcome for both craving and alcohol-drinking quantity, suggesting study characteristics can influence the assessment of treatment efficacy (Hendershot et al., 2017). More recently, other treatments such as sodium oxybate have shown significant treatment efficacy compared to placebo controls, particularly in patients with very high drinking risk level (van den Brink et al., 2018). However, one drawback to sodium oxybate for the treatment of AUD is its abuse potential since there have been reports of patients recreationally abusing it during treatment (van den Brink et al., 2018). As such, improving pharmacotherapies for AUD is necessary to reduce drinking and improve treatment outcomes.

Ketamine is a dissociative drug that acts primarily through NMDAR antagonism (Harrison and Simmonds, 1985). In the clinic, high dose ketamine [1–2 mg/kg, intravenous (i.v.)] is commonly used as an anesthetic since it has nociceptive properties and because its lack of impact on respiratory function eliminates the risk of overdose (Ivani et al., 2003). Recently, low dose ketamine (usually 0.5 mg/kg, i.v. over 40 min infusion) has shown great therapeutic benefit for patients suffering from treatment-resistant depression (TRD). Indeed, a single i.v. infusion of 0.5 mg/kg ketamine alleviates depressive-like symptoms within 2-h and has long-lasting effects for up to 2-weeks (Berman et al., 2000; Zarate et al., 2006). Furthermore, repeated low-dose ketamine infusions administered intermittently produced increased treatment response rates and sustained low-depressive scores, indicating that a repeated treatment regimen is more effective for treating TRD compared to a single ketamine infusion (Murrough et al., 2013; Shiroma et al., 2014). Following the success of low-dose ketamine for TRD, other investigators embarked on examining the potential clinical benefits of ketamine for the treatment of AUD (Yoon et al., 2019; ClinicalTrials.gov, Identifier: NCT03658330). The initiation of a clinical trial investigating ketamine as an AUD treatment option was supported by preclinical studies that previously showed acute, low-dose administration reduced alcohol intake in chronically drinking male and female rats (Sabino et al., 2013; Holleran et al., 2016; Rezvani et al., 2017, Table 1).

**Table 1:** Summary of preclinical studies investigating the effect of ketamine administration on alcohol addictive-like behaviors.

Subject	Sex	Alcohol paradigm	Ketamine paradigm	Effect	Reference
Alcohol-preferring rats	M	Operant self-administration (10% v/v)	Acute: 10 mg/kg (i.p.) Acute: 20 mg/kg (i.p.)	No change Intake	Sabino et al. (2013)
C57BL6 mice	F	CA2BC10% + 2-week withdrawal	Acute: 3 mg/kg (i.p.)	Anxiety-like behavior (NSFT)	Holleran et al. (2016)
Alcohol-preferri ng rats	M,F	CA2BC10%	Acute: 5 mg/kg (i.p.) Acute: 7.5 mg/kg (i.p.) Acute: 10 mg/kg (i.p.)	%pref (M,F) Intake/ %pref (M,F) Intake/ %pref (M,F)	Rezvani et al. (2017)
Sprague–Dawley rats	M,F	IA2BC20% (High-drinkers) IA2BC20% (Low-drinkers)	Chronic: operant self-administration 0.5 mg/kg/inf (i.v.)	Intake/ %pref (M only) Intake/ %pref (F only)	Strong et al. (2019)

*Acute, low-dose ketamine administration reduces alcohol intake and preference along with alleviating anxiety-like behavior induced by alcohol withdrawal in rodents of both sexes. Chronic operant self-administration of ketamine reduced alcohol intake and preference in male, but not female, high-drinkers while increasing alcohol intake and preference in female, but not male, low-drinkers. Abbreviations: CA2BC10%, continuous access 2-bottle choice (10% alcohol v/v); IA2BC20%, intermittent access 2-bottle choice (20% alcohol v/v); i.p., intraperitoneal; i.v., intravenous; NSFT, novelty suppressed feeding test*.

One drawback of ketamine as a potential TRD treatment is the fact that, even at low doses, it causes feelings of dissociation, depersonalization, and, at times, mild hallucinations (see review by Strong and Kabbaj, 2018). However, in both people with a family history of AUD as well as alcohol-dependent patients, the dissociative symptoms are blunted following a single i.v. infusion of ketamine at the same dose used for TRD patients (Krystal et al., 2003; Petrakis et al., 2004). These effects are likely the result of long-lasting alterations in NMDAR function in these subsets of people since similar findings have been reported with other NMDAR antagonists. For example, people with a family history of AUD were found to be less sensitive to the dissociative effects of memantine, an NMDAR antagonist, and this effect was attributed to enhanced baseline NMDAR function within these individuals (Jamadar et al., 2012). The fact that people with AUD experience fewer dissociative symptoms from ketamine compared to TRD patients suggests that ketamine as an AUD treatment option may be more efficacious given fewer side effects.

Importantly, though, the studies referenced above investigated only the effects of a single infusion of ketamine. As with depression, AUD is a lifelong disorder and would require repeated ketamine infusions to maintain long term sobriety. This is significant given that repeated exposure to ketamine may have abuse potential as demonstrated by pre-clinical reports showing that rats will self-administer ketamine at doses as low as 0.1 mg/kg/infusion (De Luca and Badiani, 2011; Wright et al., 2017; Caffino et al., 2018). Furthermore, ketamine, a “club drug” known to be taken recreationally in combination with alcohol, has abuse potential in humans and is listed as a Schedule III drug. This raises questions about the safety of treating AUD with a known addictive agent since studies have shown that polysubstance abuse is common among users of club drugs, particularly with alcohol (Wu et al., 2006, 2009). Therefore, understanding the safety of such repeated treatment regimens in both male and female subjects is necessary before using repeated ketamine administration as a viable AUD treatment option. Given the success of ketamine for the treatment of depression, possible its efficacy for AUD would be highest in individuals suffering from comorbid addiction and depression. As such, the purpose of this review is to discuss neural mechanisms of alcohol and ketamine effects under acute and chronic regimens. Here, we will highlight cell-type, brain region, and circuit-specific changes that alcohol and/or ketamine induce that are relevant in mediating their addictive and therapeutic properties. By better understanding the neurobiology of chronic alcohol and ketamine exposure, we hope through this review to gain a better understanding of the safety of repeated ketamine infusions for the treatment of AUD.

## Neural Mechanisms of Alcohol’s Addictive Properties

In general, drug addiction occurs through aberrant changes in synaptic and structural plasticity following repeated drug exposure. As such, neural mechanisms involved in acute drug exposure usually differ from those involved in chronic exposure. For alcohol, acute exposure leads, among other things, to NMDAR antagonism while chronic exposure potentiates these receptors (Lovinger et al., 1989; Floyd et al., 2003). While it seems paradoxical that a drug initially acting as an NMDAR antagonist could, over time, act as an NMDAR agonist, there are several potential mechanisms through which this might occur. The sections below will highlight alcohol-mediated changes in transmitter systems, receptors, and cell-type-specific potentiation.

The striatum is the main component of reward-related circuitry and is divided into the dorsal striatum with dorsomedial and dorsolateral subregions (DMS and DLS, respectively) and the ventral striatum that consists of the nucleus accumbens (NAc) and olfactory tubercle. Within the striatum, the principal neurons are GABAergic medium spiny neurons (MSNs) that are highly cell-type specific since 90–95% express either the dopamine 1 or 2 receptor (D1Rs or D2Rs; Gerfen et al., 1990; Le Moine et al., 1990; Lobo et al., 2006). Both MSN subtypes function through G-protein coupled processes in an opposing manner depending on their response to extracellular dopamine. D1Rs couple to stimulatory G-proteins (GαS) to facilitate the production of cyclic AMP (cAMP) and promote gene transcription. However, D2Rs couple to inhibitory G-proteins (Gαi) to inhibit cAMP production and gene transcription (Neve et al., 2004; Del’guidice et al., 2011). Within the striatum, the NAc is considered the hub of reward circuitry since it receives converging dopaminergic input from the ventral tegmental area (VTA) and glutamatergic input from regions such as the prefrontal cortex (PFC), hippocampus (HPC), and amygdala. Here, this review will focus on how acute vs. chronic alcohol exposure impacts NAc MSNs and briefly summarize the impact on DMS MSNs by summarizing how transmitter systems, receptors, and circuitry are affected.

### Acute Alcohol Exposure

Acute alcohol exposure induces a net inhibitory effect within several brain regions through bimodal actions on both NMDA receptor blockade and a reduction in glutamate release (Lovinger et al., 1989; Carboni et al., 1993; Floyd et al., 2003). Blockade of NMDARs has been demonstrated in several preclinical studies through the measurement of NMDAR-mediated excitatory postsynaptic potentials/currents (EPSPs/EPSCs) in brain regions including the hippocampus, cortex, amygdala NAc, and dorsal striatum (DS; Calton et al., 1999; Maldve et al., 2002; Yaka et al., 2003; Kolb et al., 2005; Yin et al., 2007). Furthermore, the reduction in glutamate release within the striatum following acute alcohol exposure has been demonstrated through microdialysis studies in rats (Carboni et al., 1993). Together, the interaction between reduced glutamate along with NMDAR blockade indicates a reduction in excitatory neurotransmission following acute alcohol exposure.

Extracellular dopamine is increased within the NAc following acute alcohol exposure, as demonstrated by several preclinical studies using microdialysis to measure extracellular dopamine in rodents (Yoshimoto et al., 1992; Yan, 1999; Yim and Gonzales, 2000; Vena et al., 2016). Furthermore, acute alcohol-induced dopamine release occurred exclusively in the NAc since increased extracellular dopamine levels were not observed in the dorsal striatum until alcohol was administered repeatedly (Vena et al., 2016). The increase in extracellular dopamine in the NAc was shown to be a result of increased dopamine release from the VTA rather than inhibition of dopamine reuptake within the NAc (Yim and Gonzales, 2000). The dopaminergic neurons of the VTA are under tonic control of GABAergic neurons within the same brain region, and it has shown that acute alcohol exposure depresses VTA GABAergic inhibitory postsynaptic currents (IPSCs; Xiao and Ye, 2008). Furthermore, a modulatory role of alcohol on mu-opioid receptors expressed on VTA GABAergic neurons has been shown, likely through increases in endogenous beta-endorphin levels (Méndez et al., 2001; Xiao and Ye, 2008; Jarjour et al., 2009). As a result, increased VTA dopamine release following acute alcohol exposure likely occurs, in part, as a result of enhanced opioidergic signaling on GABAergic neurons in the VTA to disinhibit nearby dopaminergic neurons. Together, these findings indicate that increased extracellular striatal dopamine activity as a result of increased VTA dopamine release mediates the rewarding effects of acute alcohol exposure.

Adenosine is a signaling molecule that is produced both intra- and extracellularly through the metabolism of adenosine triphosphate (ATP) *via* nucleotidase (Nam et al., 2013a). When produced intracellularly, it is released into the synapse through adenosine transporters, and the most prominent adenosine transporter known to play a role in alcohol’s rewarding properties is equilibrative nucleoside transporter 1 (ENT1; Nam et al., 2013a). Extracellular adenosine can then bind to adenosine receptors expressed in a cell-type-specific manner on GABAergic MSNs, with adenosine 1 receptors (A1Rs) specific to D1-MSNs and adenosine 2a receptors (A2aRs) specific to D2-MSNs in the striatum (Dixon et al., 1996; Shen et al., 2008). Studies have shown that acute alcohol exposure increases levels of extracellular adenosine by blocking its reuptake through ENT1 (Nagy et al., 1990; Krauss et al., 1993). The increased extracellular adenosine then activates A2aRs on D2-MSNs, which function as stimulatory G-protein coupled receptors (G_s_ GPCRs), in contrast to D2Rs on D2R-containing MSNs, which are G_i_ GPCRs (Shen et al., 2008). As such, G_s_-coupled D1Rs on D1R-containing MSNs and A2aRs on D2R-containing MSNs similarly stimulate intracellular signaling cascades that release intracellular stores of Ca^2+^ and activate cAMP-dependent gene transcription in the NAc (Swapna et al., 2016). Inhibitory D2 GPCRs form heteromers with A2aRs on D2R-containing MSNs and can modulate the stimulatory role of A2aRs based on the balance between extracellular adenosine and dopamine (Azdad et al., 2009; Swapna et al., 2016). Activation of the A2aR on this MSN subtype leads to the activation of adenylyl cyclase (AC) and increases the level of cyclic AMP (cAMP). Elevated cAMP activates the regulatory subunit of protein kinase A (PKA) and releases the catalytic subunit (C_α_), which translocates from the Golgi to the nucleus, and remains there until alcohol exposure ends, where it increases gene expression, specifically by phosphorylating the Ser^133^ site of CREB to initiate transcription (Nestler, 2004). Interestingly, functional studies using either ENT1-null mice or pharmacologically inhibiting ENT1 in the dorsomedial striatum have shown increased alcohol consumption as a result of increased extracellular adenosine (Choi et al., 2004; Nam et al., 2013b). Together, these results suggest that adenosine signaling on D2R-containing MSNs may be important for the acquisition of alcohol-drinking behaviors.

In the NAc, one report showed that mice exposed to a single session of binge-like alcohol drinking displayed enhanced activation of D1R-containing MSNs through recruitment of the mammalian target of rapamycin (mTOR) signaling pathway (Beckley et al., 2016). Here, it was shown that D1R stimulation was enough to activate mTOR signaling (Beckley et al., 2016). Furthermore, mTOR pathway activation on this MSN subtype in the NAc led to increased translation of the GluA1 subunit of the AMPA receptor, which is critical for the induction of long-term potentiation (LTP; Kristensen et al., 2011). The same report showed that acute alcohol intake increased the trafficking of GluA2-lacking, Ca^2+^-permeable AMPARs, thereby reducing the threshold for further potentiation of NAc D1-MSNs (Beckley et al., 2016). While acute alcohol administration is associated with reduced glutamatergic signaling, increased glutamate release in the NAc after repeated exposure to alcohol, which will be discussed below, would make D1-MSNs more susceptible to LTP induction. For example, one report identified Prosapip-1, a downstream signaling molecule of mTOR, as a target required for alcohol-dependent increases in NAc dendritic spine density and insertion of GluA2-lacking AMPARs and further showed that knockdown of Prosapip-1 in the NAc reduced alcohol self-administration in mice (Laguesse et al., 2017). Together, these studies show that acute alcohol exposure activates mTOR on NAc D1-MSNs through D1R stimulation, which activates downstream signaling cascades that make this MSN subtype more susceptible to future potentiation which is involved in alcohol intake and seeking behaviors. Overall, acute alcohol exposure appears to act on D1R-MSNs through increases in extracellular dopamine and on D2R-MSNs from increases in adenosine. Therefore, both MSN subtypes are likely involved in the initial phase of acute alcohol intake.

### Chronic Alcohol Exposure

In the shift from acute to chronic alcohol exposure, each neurotransmitter system discussed above undergoes major changes. While acute alcohol exposure was associated with ENT1 blockade to produce increased extracellular adenosine, chronic alcohol exposure leads to a desensitization of the G_s_-coupled A2aR (Gordon et al., 1986; Charness et al., 1988; Choi et al., 2004; Allen-Gipson et al., 2009), likely as the result of sustained increased levels of extracellular adenosine acting on A2aRs. In support of this, ENT1 knockout mice displayed a reduced function of the A2a receptor in the DMS, and intra-DMS infusions of an A2aR antagonist increased alcohol intake (Nam et al., 2013b). Along with receptor desensitization, other reports indicated that the adenosine transporter, ENT1, resumed normal function following chronic alcohol exposure *via* downregulation of ENT1 expression (Gordon et al., 1990; Sapru et al., 1994). As such, chronic alcohol exposure is associated with less activation of G_s_-coupled A2aRs on D2R-containing MSNs, allowing the G_i_-coupled D2R to have more of an impact on downstream signaling cascades and leading to inhibition of cAMP-PKA mediated intracellular Ca^2+^ release and gene transcription in this MSN subtype (Swapna et al., 2016). Additionally, a recent study showed that systemic administration of an adenosine analog that activates A2aRs and inhibits ENT1 reduced alcohol intake in chronically drinking mice, suggesting that while A2aR activation may play a role in the acquisition of alcohol consumption, it may be less involved in regulating the maintenance of alcohol intake (Hong et al., 2019).

In people with a family history of AUD, the expectation of an alcohol reward is associated with increased dopamine release into the NAc (Kegeles et al., 2018). However, dopamine release in the NAc is decreased in AUD patients undergoing withdrawal (Volkow et al., 2007). This is in line with data from rats which showed that alcohol cue-seeking was associated with increased dopamine release in the NAc core and dorsolateral striatum but that chronic alcohol drinking itself did not increase dopamine release (Shnitko and Robinson, 2015). One study examining dopamine’s role in associative learning in monkeys found that when exposed to a cue before a rewarding stimulus on the first trial, dopamine neurons increased firing following stimulus presentation as compared to the cue. Over time, however, these neurons began firing during the presentation of the cue, which always preceded the stimulus, and not for the reward itself (Fiorillo et al., 2003). This study is in line with what has been shown with alcohol; acute exposure increases dopamine release in the NAc while dopamine following chronic alcohol consumption increases more for alcohol-related cues. Nonetheless, dopamine release during alcohol-seeking behaviors would have stimulatory effects on D1R-containing MSNs and inhibitory effects on D2R-containing MSNs, inducing a net excitatory effect within the striatum. In one report examining cocaine, dopamine-mediated activation of D1R-containing NAc MSNs increased ΔfosB, a transcription factor considered a marker for addiction, through a feedforward loop involving calcium calmodulin II alpha (CaMKIIα) autophosphorylation of the FosB gene (Robison et al., 2013). Similarly, ΔfosB expression in the NAc is increased in D1R- but not D2R-containing MSNs following chronic alcohol intake and this may be mediated by dopamine’s actions on D1R-containing MSNs (Lobo et al., 2013).

**Figure 1:**
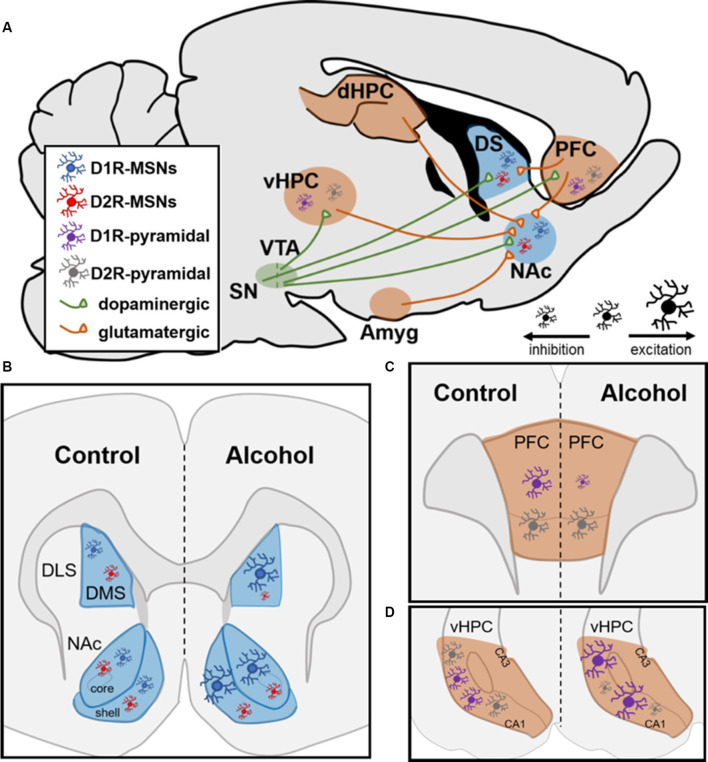
Neural circuitry and dopamine receptor-containing cells are impacted by chronic alcohol intake in rodents. **(A)** Neural circuits impacted by chronic alcohol consumption. SN, substantia nigra; VTA, ventral tegmental area; vHPC and dHPC, ventral and dorsal hippocampus; Amyg, amygdala; DS, dorsal striatum; NAc, nucleus accumbens; PFC, prefrontal cortex; MSN, medium spiny neuron. **(B,C)** Schematics showing changes in cell-type-specific excitability following chronic alcohol consumption in D1R- and D2R-MSNs in striatum and D1R- and D2R-containing pyramidal neurons in PFC and HPC. **(B)** In dorsomedial striatum (DMS) and NAc, D1R-MSNs show enhanced excitability following chronic alcohol. Alcohol increases inhibition of D2R-MSNs in DMS and does not alter them in NAc; DLS, dorsolateral striatum. **(C)** In PFC, chronic alcohol reduces the excitability of pyramidal neurons with D1Rs and does not impact cells with D2Rs. **(D)** In vHPC, chronic alcohol leads to increased excitability of pyramidal neurons containing D1Rs and increased inhibition of pyramidal neurons with D2Rs.

While acute alcohol reduces glutamate release and NMDAR-mediated EPSCs, chronic alcohol exposure is known to induce NMDAR-mediated plasticity exclusively on D1R-containing MSNs (Ji et al., 2017; Renteria et al., 2017, 2018). Electrophysiological studies have shown that chronic alcohol exposure leads to increased glutamatergic transmission within the NAc from the PFC, HPC, and amygdala (Figure 1A; Ji et al., 2017). Within the NAc, multiple reports have shown that chronic alcohol exposure led to increased firing of D1R-containing MSNs while not affecting D2R-containing MSNs in both the core and shell (Figure 1B; Renteria et al., 2017, 2018). These findings further showed increased NMDAR function on D1R- but not D2R-containing NAc MSNs, suggesting that increased NMDAR potentiation may drive the observed increases in neuronal firing (Renteria et al., 2017). Additionally, a recent report chemogenetically manipulated NAc D1R- vs. D2R-MSNs and showed that activation of D1R-containing NAc MSNs increased alcohol intake while inhibition of this MSN subtype decreased alcohol intake (Strong et al., unpublished). Importantly, these findings were extended to female rats for the first time (Strong et al., 2020). Additionally, activating D2R-containing NAc MSNs did not affect alcohol intake but inhibiting these MSNs increased intake in both sexes, suggesting a potential indirect compensatory activation of NAc D1R-containing MSNs as a result of inhibiting D2R-containing MSNs (Strong et al., 2020).

While it appears that D1R-containing MSNs within the NAc control alcohol intake, it remains unclear what intracellular signaling mechanisms contribute to these changes. As mentioned above, acute alcohol exposure led to mTOR pathway activation on D1R-containing neurons (Beckley et al., 2016). Following chronic alcohol intake, intra-NAc infusions of rapamycin, an mTOR antagonist, reduced alcohol intake in chronically drinking male mice (Cozzoli et al., 2016). While this study did not examine whether these effects were specific to D1R-containing MSNs within the NAc, it is clear that mTOR plays a regulatory role in alcohol intake in males. Interestingly, the same study showed that this is not the case in female mice since their alcohol intake was unaffected across a wide range of rapamycin doses, suggesting that mTOR may be a key regulator of alcohol’s addictive properties in male but not female mice (Cozzoli et al., 2016). However, intra-NAc infusions of MTEP, a mGluR5 antagonist, did reduce alcohol intake in both male and female mice (Cozzoli et al., 2014). Together, these studies along with those mentioned above indicate sex similarities at the cellular and receptor level induced by alcohol, but differences in recruitment of intracellular signaling pathways. Interestingly, a study examining genetic alterations in alcoholic humans observed a sex-specific polymorphism of PI3K, a kinase that activates the mTOR signaling pathway, in males but not females, supporting the finding in the study mentioned above (Desrivieres et al., 2008). Future studies should examine downstream signaling pathways from mGluR5 involved in modulating alcohol intake in male and female subjects to better understand these sex differences. Furthermore, information about whether mTOR and mGluR5 activation are specific to D1R- or D2R-containing NAc MSNs is necessary to better understand mechanisms underlying alcohol’s addictive properties.

Within the dorsomedial striatum (DMS), chronic alcohol intake led to increased glutamatergic transmission on D1R-containing MSNs and enhanced GABAergic transmission on D2R-containing MSNs (Figure 5B; Cheng et al., 2017). The same study showed that chemogenetic inhibition of D1R- and activation of D2R-containing MSNs reduced alcohol intake whereas activation of D1R- and inhibition of D2R-containing MSNs increased alcohol intake in male mice, suggesting that DMS MSNs bidirectionally control alcohol intake in a cell-type-specific manner (Figure 5B; Cheng et al., 2017). Within D1R-containing DMS MSNs, several reports have shown increased NMDAR potentiation through increased trafficking of the GluN2B regulatory subunit, which has increased conductance and channel open probability as mentioned above (Traynelis et al., 2010; Morisot and Ron, 2017). Previous reports indicate that increased GluN2B insertion enhances the activity of the NMDAR through phosphorylation of Fyn kinase (Trepanier et al., 2012). It has been shown that chronic exposure to alcohol leads to Fyn autophosphorylation in DMS through a PKA-mediated mechanism; PKA was shown to phosphorylate striatal enriched protein tyrosine phosphatase (STEP), which is a Fyn phosphatase (Wang et al., 2007; Gibb et al., 2011; Darcq et al., 2014). As a result of never getting dephosphorylated by STEP, Fyn continuously phosphorylates the GluN2B subunit of the NMDAR, which increases the channel’s open probability and, thus, increasingly potentiates the cell over time (Morisot and Ron, 2017). Recently, these findings were extended to D1R-containing neurons in the DMS since it was shown that alcohol’s enhanced glutamatergic transmission on these MSNs resulted from increased potentiation of NMDARs through the GluN2B subunit (Cheng et al., 2017). This same report suggested that the enhanced GABAergic transmission on D2R-containing DMS MSNs resulted from increased activity of the GABA_A_ receptor on these MSNs (Cheng et al., 2017). Together, these studies provide evidence for how DMS MSNs control alcohol intake in a cell-type-specific manner.

As mentioned above, PFC-NAc and HPC-NAc circuitry is impacted by chronic alcohol since these circuits display enhanced glutamatergic transmission compared to controls (Ji et al., 2017). One report using optogenetics showed that medial PFC (mPFC) to NAc core circuitry is involved in aversion-resistant alcohol intake but not alcohol intake in the absence of aversion (Seif et al., 2013). However, a recent study functionally ablating PFC glutamatergic projections to NAc using Diptheria toxin receptors showed that mPFC-NAc is necessary for cue-induced reinstatement of alcohol-seeking since blocking this circuitry abolished responding for alcohol-related cues (Keistler et al., 2017). Furthermore, Fos-positive nuclei in the mPFC increased during periods of alcohol abstinence, but not during periods of drinking, though the number of Fos-positive nuclei was shown to predict future alcohol-drinking amounts (George et al., 2012). Altogether, this data suggests that PFC-NAc circuitry may be less involved in alcohol intake itself and more involved in aversion-resistance to alcohol as well as reinstatement to alcohol-related cues. To date, no studies functionally manipulating HPC-NAc circuitry in the context of alcohol drinking exist. However, a recent study examining ventral hippocampus (vHPC) projections onto NAc shell D1R-containing MSNs showed that alcohol intake amount negatively correlated with LTD-induction, suggesting that increased alcohol intake is correlated with enhanced potentiation of NAc D1R-containing MSNs receiving inputs from vHPC (Kircher et al., 2019). The same study showed increased glutamate release from vHPC onto D1R-containing neurons in the NAc shell and the insertion of GluA2-lacking, Ca^2+^-permeable AMPARs, though these effects were not correlated with the intake (Kircher et al., 2019). Studies functionally manipulating HPC-NAc circuitry are necessary to better understand the role this circuit plays in alcohol-drinking behaviors.

Along with NAc and dorsal striatum, subregions of the PFC and HPC contain excitatory pyramidal neurons that have a cell-type-specific expression of D1Rs and D2Rs (Wei et al., 2018). A recent report showed that within the vHPC, 90% of pyramidal neurons contained either D1Rs or D2Rs (Wei et al., 2018). Additionally, in the orbitofrontal cortex (OFC) of the PFC along with the dorsomedial PFC, 85% of pyramidal neurons contain D1Rs or D2Rs in a cell-type-specific manner (Wei et al., 2018). Like the NAc, both the vHPC and the PFC receive dopaminergic input from the VTA (Lisman and Grace, 2005). In contrast to what studies have reported within the striatum, D1R-containing neurons within the PFC experience reduced firing following chronic alcohol intake whereas D2R-containing neurons are unchanged by alcohol (Figure 1C; Trantham-Davidson et al., 2017). Along with these findings, a recent report found reduced glutamatergic excitatory neurotransmission from the OFC onto D1R-containing MSNs within the DMS while there was no change in glutamatergic excitatory input from these neurons to D2R-containing MSNs (Figure 1A; Renteria et al., 2018). This may suggest that the PFC-striatal circuitry is not a major contributing factor in the heightened excitability in D1R-containing neurons within the NAc and DMS following chronic alcohol intake. However, in chronically drinking male mice, pyramidal neurons in the vHPC containing D1Rs displayed increased firing whereas those with D2Rs displayed significant reductions in firing (Figure 1D; Wei et al., 2018). This is of interest given that the vHPC monosynaptically innervates NAc, especially the NAc shell. A recent report showed that roughly 35% of vHPC-NAc inputs were to D1R-containing MSNs within the NAc shell and 40% were to D2R-containing MSNs (Li et al., 2018). Given that HPC to NAc glutamatergic transmission is heightened following chronic alcohol exposure, it is reasonable that these inputs could be impacting the NAc in a cell-type-specific manner (Ji et al., 2017; Li et al., 2018). In the future, it would be interesting to examine vHPC-NAc circuitry to better understand if it is a primary contributor to the enhanced glutamatergic input on D1R-containing MSNs following chronic alcohol intake.

## Ketamine as a Potential AUD Treatment Option

Clinical studies first demonstrated ketamine’s therapeutic benefits by showing that slow infusions of sub-anesthetic ketamine (0.5 mg/kg over 40 min) alleviated depressive symptoms in patients suffering from TRD (Berman et al., 2000; Zarate et al., 2006). However, ketamine is a schedule III drug with great potential for abuse and dependence in humans (Narendran et al., 2005; Chang et al., 2016; Schak et al., 2016). Importantly, while ketamine has abuse potential, the low doses used clinically do not generally produce strong psychomimetic effects that may underly ketamine’s rewarding properties (Zarate et al., 2006; Murrough et al., 2013). Furthermore, people suffering from AUD along with those who have a family history of AUD do not experience ketamine’s psychomimetic effects to the same degree as healthy controls, suggesting low-dose ketamine carry a lower abuse potential among these individuals (Krystal et al., 2003; Petrakis et al., 2004). Preclinical studies demonstrated ketamine’s potential therapeutic effects on alcohol intake by showing that acute, low-dose ketamine administration (≤10 mg/kg, i.p.) attenuates alcohol intake in rats of both sexes (Sabino et al., 2013; Holleran et al., 2016; Rezvani et al., 2017, Table 1). Following these findings, a clinical trial was initiated to investigate the effect of repeated intermittent ketamine administration (0.5 mg/kg, i.v. over 40 min) in people suffering from AUD (Yoon et al., 2019; ClinicalTrials.gov, Identifier: NCT03658330).

While acute administration of ketamine at low doses may have a low potential for abuse, preclinical studies have demonstrated ketamine’s abuse potential after chronic exposure by showing that rats display conditioned place preference and behavioral sensitization after repeated exposure (≤10 mg/kg, i.p.; Strong et al., 2017; Schoepfer et al., 2019). Furthermore, rats will self-administer both low- and high-dose ketamine (De Luca and Badiani, 2011; Wright et al., 2017). Ketamine’s dissociative effects have been shown to work primarily through NMDAR antagonism. In an early *ex vivo* study, a high concentration of ketamine (100 μM) applied to cerebral cortex slices abolished action potential firing* ex vivo*, through blockade of NMDARs (Harrison and Simmonds, 1985). A more recent study showed that this same ketamine concentration, when applied to primary cortical neurons, led to elevated levels of intracellular Ca^2+^ release 10-min after application with effects sustained through 1-h (Zuo et al., 2017). Eventually, the sustained elevated levels of intracellular Ca^2+^ release following ketamine had apoptotic effects, suggesting that ketamine, at high concentrations, has excitotoxic effects (Zuo et al., 2017). However, the same report showed that a low concentration of ketamine (10 μM) did not induce the excitotoxic apoptotic effects observed with the high concentration, suggesting that exposure to higher doses of ketamine may induce secondary effects unrelated to its therapeutic effect (Zuo et al., 2017). Ketamine’s antidepressant effect is exclusive to low doses (≤5 mg/kg, i.p.) in rodents since higher doses (≥20 mg/kg, i.p.) did not elicit an antidepressant response (Kim and Monteggia, 2020). Together, these studies show that ketamine as a therapeutic option might be safer and more efficacious at low doses, but this has been taken with caution as repeated and relatively higher doses of ketamine may have secondary effects contributing to excitotoxicity and abuse potential. It thus remains unclear whether repeated exposure to low-dose ketamine may induce neuroadaptations like those observed at higher concentrations. Therefore, this next section of the review will summarize what is known about neural mechanisms involved in mediating ketamine’s therapeutic vs. its addictive properties.

### Mechanisms of Ketamine’s Effects

As described above, preclinical studies have shown that acute alcohol exposure leads to the inhibition of NMDA receptors (NMDARs), which bind the excitatory neurotransmitter, glutamate (Charness et al., 1988). Conversely, chronic alcohol exposure leads to increased potentiation of NMDARs, which contributes to LTP within reward-related brain regions (Dildy and Leslie, 1989; Renteria et al., 2017; Roberto and Varodayan, 2017). NMDAR-mediated induction of LTP following chronic alcohol intake is significant given that it has been shown to causally control alcohol-seeking behaviors measured during operant alcohol self-administration (Ma et al., 2018). Along these lines, human studies have shown an increase in the number of NMDARs in the brains of humans with AUD. An early study showed an increased density of NMDARs in the PFC, a brain region critically involved in reward signaling, of alcohol-dependent brains vs. controls (Freund and Anderson, 1996). More recently, postmortem human studies examining the brains of people with AUD compared to healthy controls showed increased mRNA expression of Grin2B, the gene encoding GluN2B subunit of the NMDAR, in both the PFC and hippocampus (HPC; Zhou et al., 2011; Farris and Mayfield, 2014). This is significant given that the presence of the GluN2B subunit enhances NMDAR channel function by increasing calcium (Ca^2+^)-permeability and, thus, promotes the formation of LTP through increased synaptic strengthening (Trepanier et al., 2012; Morisot and Ron, 2017). Altogether, these studies suggest that NMDARs may be prime targets for treating people with AUD.

### Acute Ketamine Exposure

Preclinical studies examining acute, low-dose (≤10 mg/kg, i.p.) administration in rodents have shown that ketamine’s antidepressant effects are mediated through NMDAR antagonism. An initial report showed that ketamine’s antidepressant effects are associated with increased synthesis of brain-derived neurotrophic factor (BDNF), which required the inhibition of CaMKIII, also known as eukaryotic elongation factor 2 (eEF2) in the HPC (Autry et al., 2011). A follow-up study showed that eEF2 inactivation was a result of ketamine suppressing spontaneous NMDAR-mediated transmission in hippocampal slices, suggesting ketamine’s antidepressant effects depend on NMDAR antagonism (Nosyreva et al., 2013). Here, it was further shown that after ketamine’s suppression of NMDAR-mediated transmission ended, rapid synaptic potentiation associated with increased AMPAR insertion to the synapse occurred, implicating glutamatergic transmission as a primary target of ketamine (Nosyreva et al., 2013). However, a recent study suggested ketamine’s antidepressant effects might be independent of its actions on NMDARs since hydroxynorketamine (HNK), a ketamine metabolite, exerted antidepressant effects independently of NMDARs (Zanos et al., 2016). In line with early reports, though, sustained potentiation of AMPARs was necessary for the antidepressant effects of HNK (Zanos et al., 2016). Another study however has shown that HNK does block NMDARs and that the dose of HNK used in Zanos et al. (2016) was insufficient to efficiently block NMDARs (Suzuki et al., 2000). While there is a controversy on the role of NMDA receptors in mediating ketamine’s antidepressant effects, most studies agree that ketamine’s antidepressant effects are mediated through potentiation of AMPA current in the hippocampus and PFC (Autry et al., 2011; Sarkar and Kabbaj, 2016; Zanos et al., 2016).

While ketamine impacts glutamatergic transmission directly in the HPC, these effects are indirect in the mPFC given that, here, ketamine acts directly on GABAergic transmission. Reports have implicated PFC GABAergic interneurons, specifically somatostatin-expressing (SST) interneurons, in controlling ketamine’s antidepressant effects (Fuchs et al., 2017; Ali et al., 2020; Gerhard et al., 2020). Within the PFC, SST interneurons act as a microcircuit to provide inhibitory control over glutamatergic excitatory pyramidal neurons (Kepecs and Fischell, 2014). Interestingly, PFC SST interneurons are implicated in depression given that postmortem human studies showed reduced SST content in patients with depression (Sibille et al., 2011; Tripp et al., 2011; Seney et al., 2015). Furthermore, it has been shown that the disinhibition of PFC pyramidal neurons through knockdown of GABA_A_ receptors on SST interneurons produced antidepressant and anxiolytic effects in mice, indicating that SST interneurons may be a prime target for antidepressant effects (Fuchs et al., 2017). A recent study used calcium (Ca^2+^) imaging to show that ketamine inhibited SST interneurons in the mPFC, which led to the disinhibition of mPFC pyramidal neurons and enhanced glutamatergic transmission (Ali et al., 2020). Importantly, two reports have shown that ketamine’s effect on SST inhibition was through NMDAR antagonism since it was dependent upon the GluN2B subunit of the NMDAR (Ali et al., 2020; Gerhard et al., 2020). Blocking SST neurons in the PFC led to enhanced Ca^2+^ transients on PFC pyramidal neurons, suggesting that ketamine may exert NMDAR antagonism on inhibitory interneurons to disinhibit and induce synaptic potentiation within excitatory neurons (Ali et al., 2020). This is in agreeance with Fuchs et al. (2017) report showing that SST inhibition in mPFC produces antidepressant effects. Along these lines, studies have shown that acute ketamine administration activates the mTOR signaling pathway within the mPFC, which is associated with cell growth and survival, in male and female rodents (Li et al., 2010; Carrier and Kabbaj, 2013; Dossat et al., 2018). Activation of the mTOR signaling pathway was associated with increased expression of GluA1, Synapsin1, and PSD95, which are all protein markers highly correlated with dendritic spine number and/or head size, 2-h after ketamine with effects lasting up to 3-days (Li et al., 2010). Furthermore, dendritic spines within the mPFC increased following ketamine administration and one study showed that ketamine rescued stress-induced deficits in mPFC dendritic spines in male but not female rats (Li et al., 2010; Sarkar and Kabbaj, 2016). Taken together, ketamine likely disinhibits mPFC pyramidal neurons through blockade of NMDARs on SST interneurons, which increases the synaptic potentiation of pyramidal neurons by increasing mTOR pathway activation and induction of structural plasticity that may be behind the therapeutic effects of this drug.

It is worth noting that most studies mentioned above only investigated ketamine’s antidepressant effects in male subjects. Several reports suggest an enhanced behavioral sensitivity to ketamine’s antidepressant effects (Carrier and Kabbaj, 2013; Franceschelli et al., 2015; Sarkar and Kabbaj, 2016; Dossat et al., 2018). In the mPFC, mTOR phosphorylation was present in rats and mice of both sexes after ketamine administration, though females displayed this change at a lower dose of ketamine compared to males (Carrier and Kabbaj, 2013; Dossat et al., 2018). Similar changes were found with Akt activation in the mPFC, which occurred at a dose in female mice that were sub-threshold for males (Dossat et al., 2018). Given that phosphorylated Akt (p-Akt) can be modulated both by estrogen receptors along with BDNF-mediated activation of tyrosine kinase B (TrkB) receptors, these two receptor systems may play a synergistic role in the antidepressant effects of acute ketamine administration that would heighten sensitivity in females (Dossat et al., 2018). Furthermore, ketamine application to cultured induced pluripotent stem cell (iPSC)-derived astrocyte progenitors has been shown to bind estrogen receptor alpha (ERa) directly, and that estrogen and ketamine in combination produce additive effects on the induction of AMPAR expression (Ho et al., 2018). In line with this, ovariectomized female rats do not display antidepressant-like responses to acute ketamine administration until supplemented with estrogen and progesterone, suggesting a role for ovarian hormones in modulating ketamine’s antidepressant effects (Carrier and Kabbaj, 2013). Similar findings with p-Akt were observed in the HPC along with elevated activation of CaMKIIα, which can promote the induction of plasticity through AMPAR trafficking to the synapse (Lu et al., 2010; Dossat et al., 2018). The sex differences described above may arise from a heightened sensitivity to ketamine’s antidepressant effects in female rodents. Sex differences in ketamine metabolism have been described in a recent study that showed significantly greater levels of norketamine (NK) and dehydronorketamine (DHNK), ketamine metabolites, in the plasma of female rats as compared to males (Saland and Kabbaj, 2018). Furthermore, significantly greater concentrations of ketamine and NK were found in the PFC and HPC of female rats compared to males, indicating a slower clearance rate and longer half-life of ketamine in female rats (Saland and Kabbaj, 2018). Given that male and female subjects display sex differences in the neural mechanisms mediating ketamine’s therapeutic properties, both male and female subjects must be taken into consideration when investigating the safety of ketamine as a therapeutic agent.

Furthermore, acute ketamine exposure may have rewarding effects. While acute ketamine impacts both glutamatergic and GABAergic neurotransmission to mediate antidepressant effects, it also affects the dopaminergic transmission, which is involved in mediating both its rewarding and potential addictive properties. A recent meta-analysis examining studies testing acute, low-dose ketamine’s effect on dopamine release found significantly increased dopamine release in both mPFC and NAc of rodents following a single exposure (Kokkinou et al., 2018). As such, it is critical to understand how these neural mechanisms change following repeated ketamine exposure given that AUD patients would receive repeated infusions.

### Chronic Ketamine Exposure

Behavioral studies show that repeated, low-dose ketamine administration induces sensitization to its locomotor activating effects in male and female rats (Strong et al., 2017; Schoepfer et al., 2019). Additionally, ketamine sensitization was associated with increased dendritic spine density in the NAc, suggesting the induction of structural plasticity alterations within a reward-related brain region in both sexes (Strong et al., 2017). Ketamine sensitization was also associated with elevated levels of ΔfosB in the NAc of both sexes (Strong et al., 2017; Schoepfer et al., 2019). The induction of ΔfosB and structural plasticity suggests enhanced glutamatergic transmission within the NAc following repeated ketamine exposure.

This increased excitatory neurotransmission within the NAc suggests a potential shift from acute NMDAR antagonism to chronic NMDAR potentiation following chronic ketamine administration, which is a similar observation to alcohol’s mechanism of action. While it remains unknown how the shift from NMDAR antagonism to potentiation within the NAc might occur, there is some evidence that suggests it. Indeed, ketamine intravenous self-administration was associated with increased autophosphorylation of CaMKIIα in the NAc of male rats, specifically increased phosphorylation of CaMKIIα at the Thr286 site, which is associated with the induction of LTP and learning (Giese et al., 1998; Caffino et al., 2018). It is worth noting that a previous report examining cocaine’s addictive effects showed that CaMKIIα autophosphorylation in the NAc continuously increased ΔfosB expression and triggered CREB-mediated transcription (Robison et al., 2013).

Ketamine self-administration was also associated with elevated phosphorylation of the GluN2B subunit of the NMDAR in male rats, suggesting potentiation of this NMDAR subunit may also control ketamine’s addictive properties (Caffino et al., 2018). Additionally, ketamine self-administration led to a reduction in BDNF and p-Akt within the NAc suggesting a potential reduction in NAc mTOR signaling in male rats (Caffino et al., 2016). Within the Akt pathway, phosphoinositide 3-kinase (PI3K) is upstream while mTOR is downstream and feedback loops exist such that mTOR can get indirectly turned on or off (Carracedo and Pandolfi, 2008). In a positive feedback loop, Akt activates nuclear factor kappa beta (NFκB) which turns off PI3K to stop mTOR activation and maintain cell health (Carracedo and Pandolfi, 2008). This is of interest given that a separate report revealed that a low concentration of ketamine (10 μM) application to cultured neurons led to the translocation of inactive NFκB in the cytoplasm to its active state in the nucleus following elevated levels of intracellular Ca^2+^ release (Wang et al., 2006). In the nucleus, activated NFκB triggers transcription and, recently, the ENCODE project revealed that transcription of the GluN2B subunit is NFκB-dependent (Xu and Lipsky, 2015). It is therefore plausible that ketamine-induced reductions in BDNF and p-Akt activation could explain increased GluN2B insertion within the NAc. Furthermore, ketamine sensitization was associated with increased GluA1 in the NAc of male and female rats, suggesting increased AMPAR insertion as well (Strong et al., 2017). Both the increased AMPAR expression and the GluN2B activation could explain a shift to NMDAR potentiation after repeated ketamine exposure, though future studies will need to confirm this. Importantly, alcohol’s addictive properties are controlled by GluN2B activation on D1R-containing MSNs, indicating the mechanism by which alcohol and ketamine’s rewarding properties are mediated might have some overlap (Cheng et al., 2017; Morisot and Ron, 2017).

To date, however, no studies have examined whether ketamine’s addictive effects are controlled by D1R- or D2R-containing MSNs in reward-related brain regions, but pharmacological studies suggest that both D1Rs and D2Rs may play a role. For instance, acute ketamine administration has been shown to recruit both D1 and D2Rs given that intra-NAc D1R antagonism was abolished while D2R-antagonism attenuated ketamine-induced increases in locomotor movement (Matulewicz et al., 2010). Another report indicated that hippocampal evoked field potentials in the NAc were suppressed by ketamine and while D2R antagonism rescued these effects, D1R antagonism did not (Hunt et al., 2005). These findings should be taken lightly though, since pharmacological manipulations affect presynaptic NAc dopamine receptors as well, which are not cell-type specific.

Though evidence for a mechanism of action involved in controlling ketamine’s addictive properties is limited, the currently available evidence highlights potential pathways involved. Like alcohol, repeated exposure to ketamine provides evidence of increased glutamatergic transmission based on increases in dendritic spines, ΔfosB expression, CaMKIIα autophosphorylation, and increased phosphorylation of the GluN2B subunit of the NMDAR. Additionally, ketamine may inhibit mTOR signaling given reduced BDNF and p-Akt in the NAc of male rats. Given that alcohol intake can be controlled by inhibiting mTOR in the NAc of male but not female mice, it is possible that if ketamine’s therapeutic effects on alcohol intake involve mTOR that treatment for AUD would be more beneficial for male compared to female subjects. One study showed that ketamine-induced reductions in alcohol intake require mTOR signaling since rapamycin blocked these effects (Sabino et al., 2013).

As described above, ketamine induces a shift from NMDAR antagonism to potentiation of these receptors’ function. One possible way this might occur could be through low dose ketamine antagonizing NMDAR located on GABA parvalbumin neurons within the mPFC, which disinhibit excitatory pyramidal neurons that directly project to the NAc (Ali et al., 2020). If enhanced excitation within the mPFC led to increased glutamate release within the NAc following repeated ketamine exposure, neuroadaptations could occur that parallel structural and synaptic alterations seen with other drugs of abuse. In general, alcohol and ketamine’s mechanism of action overlap in many ways, and this calls into question whether repeated ketamine infusions should be used as a treatment for AUD. Furthermore, behavioral studies indicate female rats display enhanced sensitivity to ketamine’s addictive effects, suggesting that ketamine’s therapeutic dose may be different between the sexes (Strong et al., 2017, 2019; Wright et al., 2017, 2019; Schoepfer et al., 2019). Additionally, because mechanisms resulting from repeated ketamine exposure share similarities with those of alcohol, it is critical that more steps be taken in understanding the interaction between alcohol and ketamine before its use as an AUD treatment option in humans. However, repeated ketamine infusions for AUD patients may be administered safely if only a certain number of infusions are received. One report showed that four i.v. infusions of low-dose ketamine did not alter vulnerability to ketamine addiction, demonstrated by the fact that neither male nor female rats increased ketamine self-administration after receiving therapeutic infusions (Wright et al., 2019).

## Interactions Between Alcohol and Ketamine

While several studies report on neural mechanisms contributing to both alcohol and ketamine’s addictive and therapeutic effects, much less is known regarding how these two drugs interact. In a recent report, ketamine self-administration (0.5 mg/kg/infusion, i.v.) reduced alcohol intake in high-alcohol drinking male but not female rats, highlighting the critical need for sex to be more thoroughly examined as a factor before clinical testing of treatment options (Strong et al., 2019). The same study found that high-alcohol intake increased NAc dendritic spine density in both sexes, and while ketamine self-administration reduced these effects in males it did not in female rats, suggesting that ketamine rescued the structural alterations in the NAc from alcohol in male but not female rats (Strong et al., 2019). It should be noted, though, that another study found that alcohol withdrawal reduced thin spine density in the NAc, though this could be due to differences in length of withdrawal and alcohol intake amount (Spiga et al., 2014). Interestingly, ketamine did not impact alcohol intake in low-alcohol drinking male rats and increased intake in low-alcohol intake female rats, indicating that individual differences in alcohol intake may also alter the response to ketamine (Strong et al., 2019).

Other studies have examined the effects of co-administration of alcohol with high doses of ketamine. In male rats, one study reported that co-administration of 20% alcohol with a high dose of ketamine (30 mg/kg, i.p.) increased VTA extracellular dopamine while ketamine on its own did not, suggesting that co-administering these drugs may amplify their rewarding properties (Zhang et al., 2018). Furthermore, tyrosine hydroxylase mRNA was increased with co-administration of alcohol and 30 mg/kg ketamine (Zhang et al., 2018). It has also been shown that co-administration of alcohol with a high concentration of ketamine (100 μM) amplified ketamine-induced apoptosis through heightened levels of intracellular Ca^2+^ (Zuo et al., 2017).

It should be noted, though, that in the clinic these two drugs will not be co-administered together since ketamine infusions will be administered to AUD patients abstinent from alcohol. Furthermore, the studies highlighted above examined high doses of ketamine while clinical studies are utilizing slow infusions of a low dose of ketamine (0.5 mg/kg, i.v. over 40 min; ClinicalTrials.gov, Identifier: NCT01558063). While no studies to date have examined the effect of repeated low dose ketamine administration on alcohol intake, a recent report showed that repeated, low-dose administration of ketamine (2.5 mg/kg, i.p.) attenuated alcohol-withdrawal induced depressive-like phenotypes in male rats (Getachew and Tizabi, 2019). Additionally, NBQX, an AMPAR antagonist, also attenuated alcohol-withdrawal induced depressive-like effects in rats, suggesting AMPARs blockade may have therapeutic benefits during alcohol withdrawal (Getachew and Tizabi, 2019). Together, these studies suggest that sex, individual differences, and the dose of ketamine can differentially alter the response to ketamine for AUD. Future studies should expand on sex and individual differences in AUD treatment response with repeated low-dose ketamine administration, and investigate neuroadaptations mediating ketamine’s therapeutic effects for AUD to better understand the safety of its use in both male and female subjects.

## Conclusion

Several reports indicate that repeated exposure to alcohol increases glutamatergic transmission through NMDAR potentiation in reward-related brain regions such as the NAc and DMS. Ketamine is being investigated as a potential treatment option given that, acutely, it acts as an NMDAR antagonist (ClinicalTrials.gov, Identifier: NCT01558063). However, given that AUD is a chronic relapsing disorder, multiple ketamine infusions are necessary to maintain sobriety. After repeated exposure, ketamine appears to also potentiate NMDARs, calling into question the safety of its use as an AUD treatment option. Furthermore, most studies have only investigated these effects in male subjects, and a recent study suggests that repeated ketamine exposure may not reduce alcohol intake in female rats the same way it does in male rats (Strong et al., 2019). Interestingly, though, the R-ketamine enantiomer has shown some therapeutic potential as a treatment for opioid use disorder in rats given that it blocked morphine-induced conditioned place preference and alleviated withdrawal symptoms (Witkin et al., 2020). While the evidence presented in this review suggests that repeated ketamine treatment for AUD may put patients at further risk for addiction-like behaviors, future studies should tease apart effects of S- vs. R-ketamine enantiomers as well as differences in males vs. females. Additionally, no studies to date have examined ketamine as a treatment option for adolescents suffering from AUD, a group comprised of around 400,000 individuals (National Institute on Alcohol Abuse and Alcoholism, 2018). This is extremely important given that adolescence is a period when drug abuse often starts and because ketamine’s therapeutic effects are exerted through mechanisms in the mPFC, which is not fully developed until adulthood (Arain et al., 2013). As such, more work is necessary for both sexes to understand the neuroadaptations occurring after repeated ketamine infusions for chronic alcohol use.

## Author Contributions

CS wrote the first draft of this review and then MK and CS worked together to edit it. All authors contributed to the article and approved the submitted version.

## Conflict of Interest

The authors declare that the research was conducted in the absence of any commercial or financial relationships that could be construed as a potential conflict of interest.
